# Sustained Transmission of Neisseria gonorrhoeae Strains with High-Level Azithromycin Resistance (MIC ≥ 256 μg/mL) in Argentina, 2018 to 2022

**DOI:** 10.1128/spectrum.00970-23

**Published:** 2023-06-20

**Authors:** Ricardo Ariel Gianecini, Tomas Poklepovich, Daniel Golparian, Noelia Cuenca, Laura Scocozza, Silvina Bergese, Liliana Fernández Canigia, Viviana Vilches, María Julia Lazzarino Elgart, Magnus Unemo, Josefina Campos, Patricia Galarza

**Affiliations:** a World Health Organization Collaborating Centre for Antimicrobial Resistance, National Reference Laboratory of Sexually Transmitted Diseases (STD), National Institute of Infectious Diseases - ANLIS “Dr. Carlos G. Malbrán”, Ciudad Autónoma de Buenos Aires, Argentina; b National Center of Genomics and Bioinformatics - ANLIS “Dr. Carlos G. Malbrán”, Ciudad Autónoma de Buenos Aires, Argentina; c World Health Organization Collaborating Centre for Gonorrhoea and Other STIs, Örebro University, Örebro, Sweden; d Laboratorio Bacteriología, Hospital Fernández, Ciudad Autónoma de Buenos Aires, Argentina; e Laboratorio Bacteriología, Hospital Alemán, Ciudad Autónoma de Buenos Aires, Argentina; f Laboratorio Microbiología, Hospital Universitario Austral, Buenos Aires, Argentina; g Laboratorio Bacteriología, Hospital Rawson, Córdoba, Argentina; h Institute for Global Health, University College London, London, United Kingdom; Riverside University Health System Medical Center, University of California

**Keywords:** gonorrhea, azithromycin, antimicrobial resistance, treatment, whole-genome sequencing

## Abstract

Azithromycin combined with ceftriaxone is the recommended dual therapy for uncomplicated gonorrhea in many countries. Nevertheless, the increasing prevalence of azithromycin resistance compromises the effectiveness of this treatment strategy. From 2018 to 2022, we collected 13 gonococcal isolates with high-level azithromycin resistance (MIC ≥ 256 μg/mL) across Argentina. Whole-genome sequencing revealed that these isolates were mainly represented by the internationally spreading Neisseria gonorrhoeae multi-antigen sequence typing (NG-MAST) genogroup G12302, containing the 23S rRNA A2059G mutation (in all four alleles) together with mosaic *mtrD* and *mtrR* promoter 2 loci. This information is important to develop targeted public health policies to control the spread of azithromycin-resistant N. gonorrhoeae in Argentina and internationally.

**IMPORTANCE** Azithromycin resistance in Neisseria gonorrhoeae has been increasing in numerous populations worldwide, which is of concern, as azithromycin is part of the recommended dual treatment in many countries. Here, we report 13 N. gonorrhoeae isolates with high-level azithromycin resistance (MIC ≥ 256 μg/mL). This study observed that high-level azithromycin-resistant gonococcal strains have shown sustained transmission in Argentina and are related to the successful international clone NG-MAST G12302. Genomic surveillance together with real-time tracing and data-sharing networks will be crucial in controlling the spread of azithromycin resistance in gonococcus.

## OBSERVATION

Antimicrobial resistance in Neisseria gonorrhoeae is a global public health concern. The extended-spectrum cephalosporin ceftriaxone is the last option for first-line empirical gonorrhea treatment, but the emergence of ceftriaxone resistance has raised concerns about future treatment (1). Consequently, ceftriaxone (250 mg−1 g) combined with azithromycin (1−2 g) is now recommended as a first-line empirical treatment for uncomplicated gonorrhea in many countries, including Argentina (2–4). In recent years, azithromycin-resistant gonococcal isolates have substantially increased internationally (1, 5, 6). Consequently, some countries have returned to ceftriaxone high-dose (0.5−1 g) monotherapy as a first-line treatment (2, 7, 8). In Argentina, a significant increase in azithromycin-resistant isolates (MICs ≥ 2 μg/mL) was recently observed (9), and high-level azithromycin-resistant (AZM-HLR) isolates (MIC ≥ 256 μg/mL) were also identified (9). This increasing azithromycin resistance threatens the effectiveness of the ceftriaxone-azithromycin dual antimicrobial treatment for gonorrhea. Here, we investigated the epidemiological and genomic characteristics of AZM-HLR N. gonorrhoeae isolates in Argentina from 2018 to 2022.

A total of 13 AZM-HLR gonococcal isolates were collected through the Gonococcal Antimicrobial Susceptibility Surveillance Program-Argentina (GASSP-AR) from 2018 to 2022. Susceptibility to azithromycin, ceftriaxone, cefixime, ciprofloxacin, spectinomycin, and gentamicin (Sigma-Aldrich, St. Louis, MO, USA) was determined by the agar dilution method and interpreted according to CLSI guidelines (10). Whole-genome sequencing (WGS) was performed on all isolates using the MiSeq platform (Illumina, San Diego, CA, USA) as previously described (9). Reads were assembled using Unicycler v0.4.8, which is based on SPAdes v3.13.0 (11). Azithromycin resistance determinants, including the 23S rRNA A2059G and C2611T mutations (Escherichia coli numbering), *mtrR*-35A, the *mtr*_120_ mutation, MtrR A39T and G45D substitutions, mosaic *mtrR*, mosaic *mtrD*, and *macAB* mutations (12, 13), were identified using a combination of *de novo* assembly and mapping-based strategies as previously described (9). Molecular typing information (N. gonorrhoeae multiantigen sequence typing [NG-MAST v2.0], multilocus sequence typing [MLST], and N. gonorrhoeae sequence typing for antimicrobial resistance [NG-STAR]) was determined using the PubMLST database (https://pubmlst.org/) (14). The NG-STAR types were also assigned to clonal complexes (CCs) (https://github.com/leosanbu/pyngoST) (15). For phylogenetic analysis, reads were mapped to the WHO-P reference genome, and single-nucleotide polymorphisms (SNPs) were identified with Snippy v4.4.5 (https://github.com/tseemann/snippy). Recombinant regions were identified and filtered using Gubbins v2.1.0 (https://github.com/sanger-pathogens/gubbins). All available genomic data from N. gonorrhoeae AZM-HLR isolates, MLST sequence type 9363 (ST9363), and NG-MAST genogroup G12302, were similarly analyzed (16–18). A maximum likelihood tree was inferred using IQ-tree v1.6.1, and the phylogeny was visualized with iTOL v6 (19, 20).

**FIG 1:**
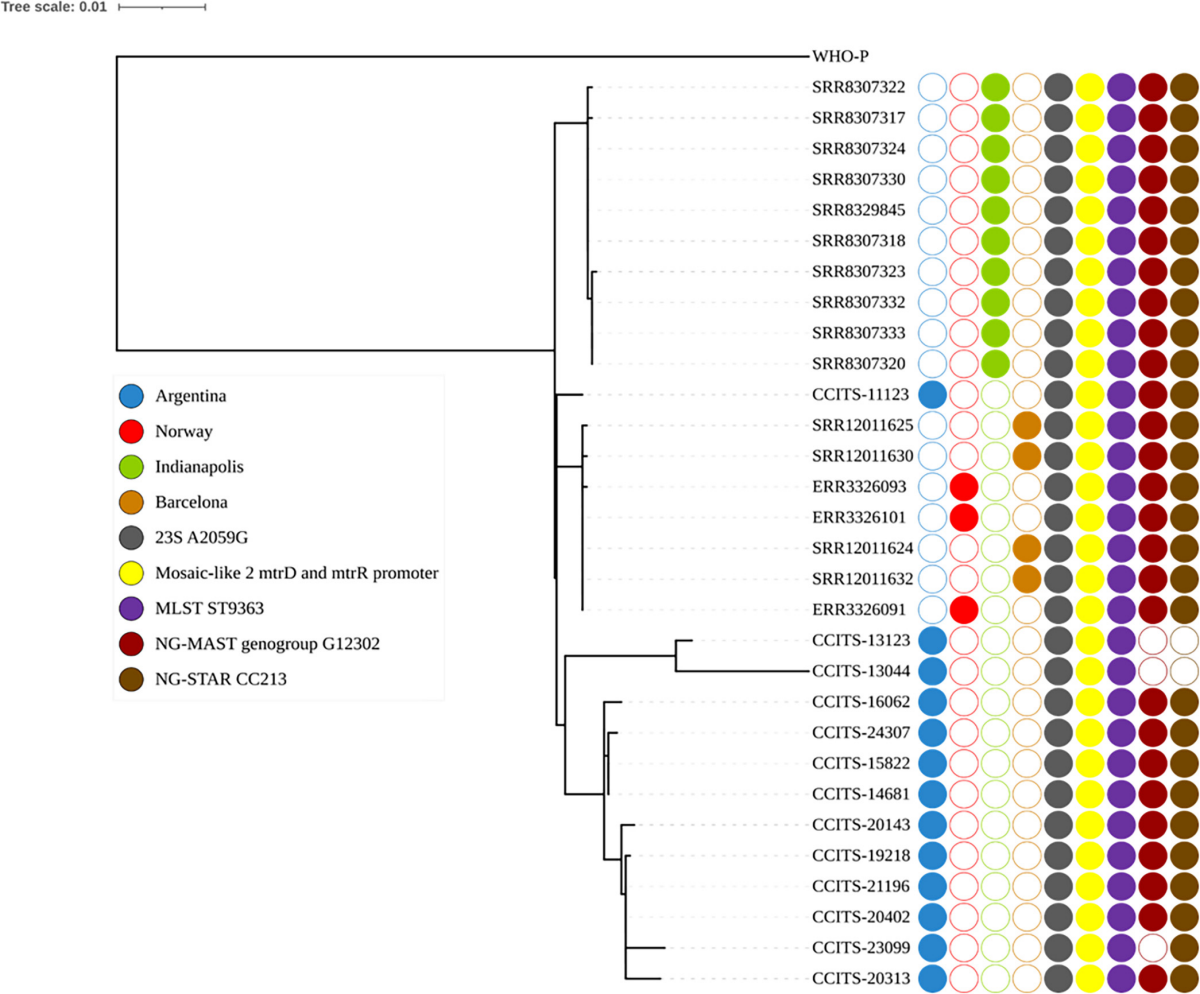
Phylogenomic tree of Neisseria gonorrhoeae isolates with high-level resistance to azithromycin (MIC ≥ 256 μg/mL) collected across Argentina (2018 to 2022), compared with isolates from Norway (2016 to 2017) (16), Indianapolis, USA (2017 to 2018) (18), and Barcelona, Spain (2017 to 2018) (17). The first four columns next to the tree represent the country or city of origin/reporting. The following two columns describe azithromycin resistance determinants and the last three columns represent the MLST ST (all ST9363), NG-MAST genogroup (all except three were G12302), and NG-STAR clonal complexes (all except two were CC213).

All patients (*n *= 13) were male, and 10 were men who have sex with men (MSM) (Table 1). The median age was 32 years (range, 20 to 54 years). Four males were HIV-positive and three had previous gonorrhea. Of the patients, 12 had urogenital infections and 1 had an extragenital infection (perianal abscess). Data regarding previous or current treatment was not available. The isolates were identified in Ciudad Autónoma de Buenos Aires (CABA; *n *= 9) and Buenos Aires (*n *= 2) and Córdoba (*n *= 2) provinces, respectively. All isolates were AZM-HLR but were susceptible to ceftriaxone, cefixime, and spectinomycin, and all except one were susceptible to ciprofloxacin (Table 1). All isolates had the 23S rRNA A2059G mutation in all four 23S rRNA gene alleles together with mosaic-like 2 *mtrR* promoter and *mtrD* sequences (5, 12) (Fig. 1). All isolates belonged to MLST ST9363, and nine were also characterized as NG-MAST ST3935 and NG-STAR type 1993 (CC213) (Table 1). The mean SNP difference between isolates was 24.1 (range, 0 to 78). The Argentinean isolates were closely related to AZM-HLR isolates from Norway, Indianapolis, USA, and Barcelona, Spain (Fig. 1) (16–18), i.e., with mean pairwise SNP differences of 26.6 (range, 12 to 64) compared to Norwegian isolates, 26.7 (12 to 64) for Barcelona isolates, and 28.9 (14 to 67) for Indianapolis isolates.

**TABLE 1:** Patient characteristics, antimicrobial susceptibility, and molecular epidemiology of Neisseria gonorrhoeae isolates with high-level resistance to azithromycin in Argentina^*a*^

Strain	Yr	Province	Gender	Sexual orientation	HIV	MIC (μg/mL)						MLST	NG-MAST type	NG-STAR type
						CRO	CFM	AZM	SPT	CIP	GEN			
CCITS-11123	2018	Buenos Aires	Male	MSM	Negative	0.016	0.008	≥256	32	0.016	8	9363	3935	1993
CCITS-13044	2019	Córdoba	Male	MSM	Positive	0.004	0.008	≥256	32	4	8	9363	20106	3194
CCITS-13123	2019	CABA	Male	Unknown	Negative	0.008	0.016	≥256	32	0.002	16	9363	20106	2906
CCITS-14681	2019	CABA	Male	MSM	Positive	0.016	0.008	≥256	32	0.016	8	9363	3935	1993
CCITS-15822	2020	CABA	Male	MSM	Negative	0.016	0.004	≥256	32	0.016	8	9363	3935	1993
CCITS-16062	2020	CABA	Male	MSM	Positive	0.008	0.008	≥256	32	0.016	8	9363	3935	1993
CCITS-19218	2021	Buenos Aires	Male	MSM	Negative	0.008	0.008	≥256	32	0.008	16	9363	3935	1993
CCITS-24307	2021	CABA	Male	Unknown	Unknown	0.008	0.016	≥256	16	0.008	8	9363	6765	1993
CCITS-20143	2022	CABA	Male	MSM	Unknown	0.008	0.008	≥256	32	0.016	8	9363	3935	1993
CCITS-20313	2022	CABA	Male	MSM	Unknown	0.008	0.008	≥256	32	0.016	8	9363	3935	1993
CCITS-20402	2022	CABA	Male	MSM	Positive	0.008	0.008	≥256	32	0.016	8	9363	3935	1993
CCITS-21196	2022	CABA	Male	Heterosexual	Negative	0.008	0.008	≥256	32	0.016	8	9363	3935	1993
CCITS-23099	2022	Córdoba	Male	MSM	Negative	0.008	0.008	≥256	32	0.002	8	9363	21175	5178

a CABA, Ciudad Autónoma de Buenos Aires; MSM, men who have sex with men; HIV, human immunodeficiency virus; CRO, ceftriaxone; CFM, cefixime; AZM, azithromycin; SPT, spectinomycin; CIP, ciprofloxacin; GEN, gentamicin.

In the present study, phylogenomic analysis showed that many AZM-HLR isolates have a high degree of genomic relatedness, suggesting a recent and sustained transmission within a sexual network mainly composed of MSMs. All isolates contained the 23S rRNA A2059G SNP, in all four alleles, which causes AZM-HLR, and an *mtrR* promoter/*mtrD* mosaic 2 encoding the MtrRCDE efflux pump system (5, 9, 12). These isolates were mainly represented by MLST ST9363 and NG-MAST ST3935, and this AZM-HLR strain appears to have been in sustained transmission at least since 2018 in Argentina (9). NG-MAST ST3935 is more than 99% similar to ST12302, which also carries an *mtrR* promoter/*mtrD* mosaic 2 sequence and has been responsible for the recent expansion of azithromycin-resistant gonococcal strains (MICs, 2 to 4 μg/mL) in Europe, Canada, and the United States (5, 6, 21). Previous gonococcal evolution studies have estimated that approximately 4 (range, 0 to 14) SNPs occur per genome per year, allowing the relationship between strains to be determined (22). Our Argentinean AZM-HLR isolates showed a high level of genomic similarity with AZM-HLR isolates reported in the United States, Spain, and Norway (mean, 26 to 29 SNPs), indicating that importation and subsequent dissemination has occurred (16–18).

In Argentina, dual therapy (ceftriaxone 500 mg plus azithromycin 1 g) is recommended as a first-line empirical treatment of uncomplicated gonorrhea (4). Nevertheless, the significant increase of low-level azithromycin resistance (MICs, 2 to 16 μg/mL) (9), and the spread of AZM-HLR isolates, may threaten the efficacy of this treatment strategy. Due to the increase of azithromycin-resistant N. gonorrhoeae isolates, some countries, such as the United States, United Kingdom and some other European countries, have returned to first-line treatment with ceftriaxone high-dose (0.5−1 g) monotherapy (2, 7, 8). High-dose ceftriaxone monotherapy for gonorrhea might be of benefit in Argentina to mitigate the selection of azithromycin-resistant isolates, which could also be reasonable because ceftriaxone resistance has been rare (23, 24). In general, there is an urgent need for improved access to N. gonorrhoeae diagnostics, antimicrobial resistance detection, and genomic epidemiological data. This information will be critical in preventing the dissemination of gonococcal clones resistant to azithromycin (and/or resistant to ceftriaxone) and preserving the current available therapeutic option for gonorrhea.

In conclusion, AZM-HLR gonococcal strains have shown sustained transmission in Argentina and are related to the successful international clone NG-MAST G12302 (5, 16–18). Enhanced antimicrobial resistance surveillance, including genomic monitoring using individual-level epidemiologic data, is necessary for the development of better-targeted public health policies to prevent the spread of these gonococcal isolates.

### Data availability.

Sequence reads for all Argentinean isolates used in this study are available at the European Nucleotide Archive (https://www.ebi.ac.uk/ena/browser/home) under the BioProject accession number PRJEB55521.
